# Advantages of cone beam computed tomography (CBCT) in the orthodontic treatment planning of cleidocranial dysplasia patients: a case report

**DOI:** 10.1186/1746-160X-7-6

**Published:** 2011-02-27

**Authors:** Domenico Dalessandri, Laura Laffranchi, Ingrid Tonni, Francesca Zotti, Maria Grazia Piancino, Corrado Paganelli, Pietro Bracco

**Affiliations:** 1 Doctoral School in "Medicine and Experimental Therapy", Specialisation in "Physiopathology of Mastication and the Stomatognathic Apparatus. Dental Materials", XXIII cycle, Department of Biomedical Sciences and Human Oncology, University of Torino, Italy; 2 Orthodontic and Gnathology - Masticatory Function Department, School of Orthodontics, University of Torino, Italy; 3 Dental School - Orthodontic Postgraduate Program - Department of Surgical Specialities, Radiological and Medical-Forensic Sciences - University of Brescia, Italy

## Abstract

Our aim was to discuss, by presenting a case, the possibilities connected to the use of a CBCT exam in the dental evaluation of patients with Cleidocranial Dysplasia (CCD), an autosomal dominant skeletal dysplasia with delayed exfoliation of deciduous and eruption of permanent teeth and multiple supernumeraries, often impacted. We think that CBCT in this patient was adequate to accurately evaluate impacted teeth position and anatomy, resulting thus useful both in the diagnostic process and in the treatment planning, with an important reduction in the radiation dose absorbed by the patient.

## Background

Cleidocranial dysplasia (CCD), also known as cleidocranial dysostosis or Marie-Sainton syndrome, is a disorder that affects most prominently those bones derived from endochondral and intramembranous ossification and it's characterized by defective development of the cranial bones and by the complete or partial absence of the clavicles. Diagnosis is based on clinical and radiographic findings, that include imaging of the cranium, thorax, pelvis and hands. Frequently these patients presents a delayed ossification of the skull fontanels and a premature closing of the coronal suture that leads to a frontal, parietal and occipital bossing of the skull; a short stature, occasionally accompanied by a spinal scoliosis; a wide and flat nasal bridge due to hypertelorism; different anomalies of pubis and hipbone, with flat feet and knock knees; a brachycephaly with an high arched palate and sometimes cleft palate; a prolonged retention of deciduous teeth and several impacted permanent successors and supernumerary elements, sometimes accompanied by follicular cysts and eruptive pseudocysts [1,2]. This pathology is transmitted as an autosomal dominant trait or it's caused by a spontaneous genetic mutation and is present at a frequency of one in one million individuals. To date, RUNX2(CBFA1) is the only gene known to be associated with CCD; although not all cases clinically diagnosed have mutations in RUNX2, there is little additional evidence for locus heterogeneity. Mutations in RUNX2 have a high penetrance and extreme variability. CCD affects all ethnic groups [3,4].

Although the spectrum of phenotypic variability in CCD ranges from primary dental anomalies to all CCD clinical features plus osteoporosis, no clear phenotype-genotype correlation has been established [5].

Children with CCD should be monitored for orthopedic complications, dental abnormalities, upper airway obstruction, sinus and ear infections, hearing loss, and osteoporosis.

### Intelligence is normal in individuals with classic CCD

The most important dental problem associated with this syndrome is the malocclusion and the crowding of the dental arches caused by the retention of multiple deciduous teeth and the presence of several supernumerary. These supernumerary, associated with a diminished alveolar bone resorption, also lead to the impaction or the ectopic location of the permanent teeth [6,7].

The previous approach to the dental problems of these patients consisted in no treatment or in the extraction of the impacted or malformed teeth and their prosthetic replacement [8,9], with a consequent important bone loss.

In the last years a more conservative approach has been developed, combining orthodontics and maxillofacial surgery. Orthodontic treatment consist in the extraction of the supernumerary teeth and the deciduous with delayed exfoliation, followed by the surgical exposure of impacted permanent teeth and their orthodontic guided eruption. Extractions are not accomplished in one time: there must be a staged approach in order to maintain the vertical occlusal dimension while the different groups of unerupted teeth are exposed and pulled in their ideal position.

If there isn't any, or just a mild, skeletal discrepancy between maxilla, mandible and cranium, the treatment is finished with the alignment of all permanent teeth, obtaining a correct occlusion and an agreeable smile aesthetics ([10].

In presence of an important skeletal discrepancy, most commonly a mandibular prognathism, that preclude the possibility to achieve an acceptable orthodontic camouflage, it's necessary to wait until the completion of skeletal growth and then restoring a correct bone position through orthognatic surgery, followed by the orthodontic finishing [11-14].

Traditional dental radiographs are very useful tools for the diagnosis of CCD, permitting to observe two features of the classical triad considered pathognomonic for diagnosis of this syndrome: multiple supernumerary teeth and open suture and fontanels of the skull (the third sign is the partial or complete absence of the clavicles). They also show other features helping in the diagnostic process like the presence of impacted teeth, the underdevelopment of maxillary sinuses and the parallelism of mandibular ramus, with an upward and posteriorly pointing coronoid process [15].

Unfortunately, especially when there are a lot of supernumerary teeth, traditional dental radiographs are not enough rich in details to allow correct planning the orthodontic treatment of patients in late mixed dentition.

In these cases it's appropriate to use a multi-slice computed tomography (MSCT) scanner with an accurate tridimensional information regarding the anatomy of every single tooth, the spatial relation between adjacent teeth and face to the surrounding anatomical structures.

These data are of crucial importance in order to perform the best orthodontic treatment for different reasons: they permit to surely identify the supernumeraries teeth, often with some hidden malformations like dilacerations or dental invagination; they give the exact position of the impacted teeth in relation to the roots of the adjacent erupted teeth, preventing damage of these roots during the forced orthodontic eruption of the impacted teeth; and they also define the position of every tooth inside the alveolar bone and near some important vascular vessels and nerve fibers, avoiding unexpected complications during the extraction or the surgical exposition of impacted teeth, like bone fracture, local hemorrhage or denervation.

Unfortunately a conventional MSCT exam expose the patient to an high dose of x-ray, thereby limiting the application of this techniques only to the most complex cases.

Recently a relatively new technique, the CBCT, reducing the dose of radiation adsorbed by the patient, had been improved by different manufacturer, obtaining good quality images. The principal difference between MSCT and CBCT is that conventional CT uses a fan of x-rays and a narrow detector, so multiple slices are stacked to obtain a complete image, whereas CBCT use a cone of x-rays and a two-dimensional square detector allowing a single rotation of the radiation source to capture an entire region of interest: hereby the total radiation is less important than with conventional CT [16].

## Case Report

**Figure 1:**
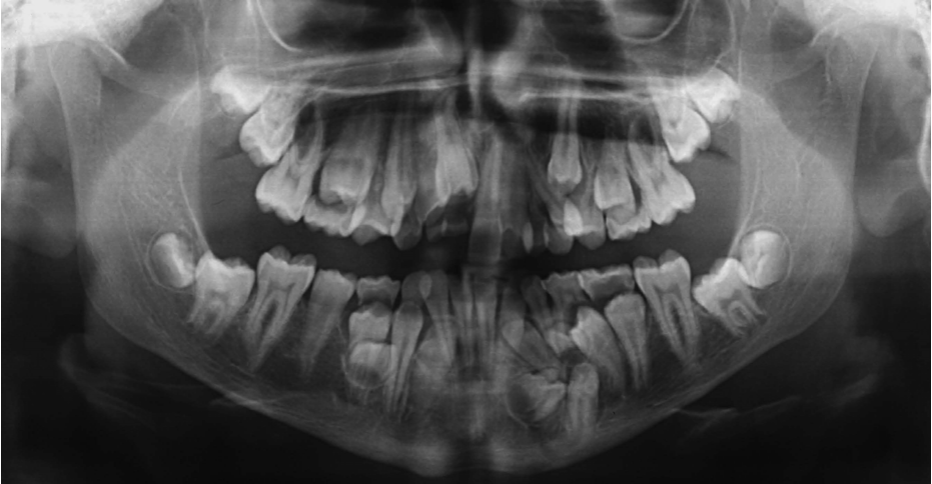
**Orthopantomography at 13 years 2 months of age**.

**Figure 2:**
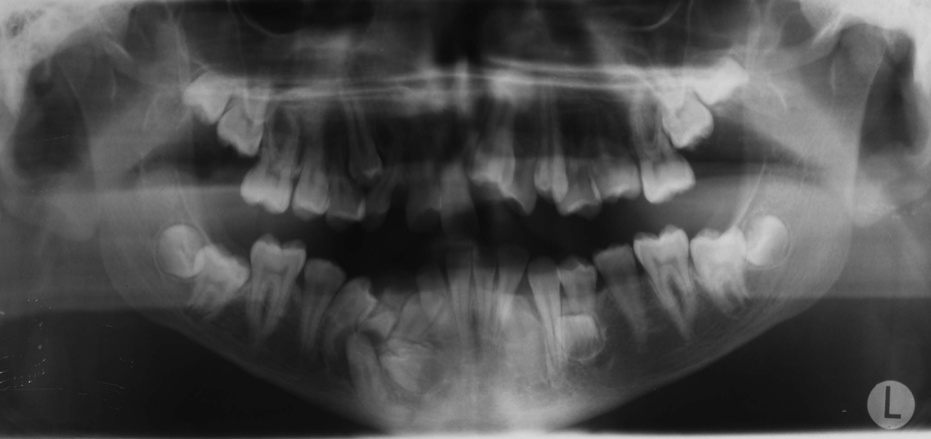
**Orthopantomography at 13 years 9 months of age**.

**Figure 3:**
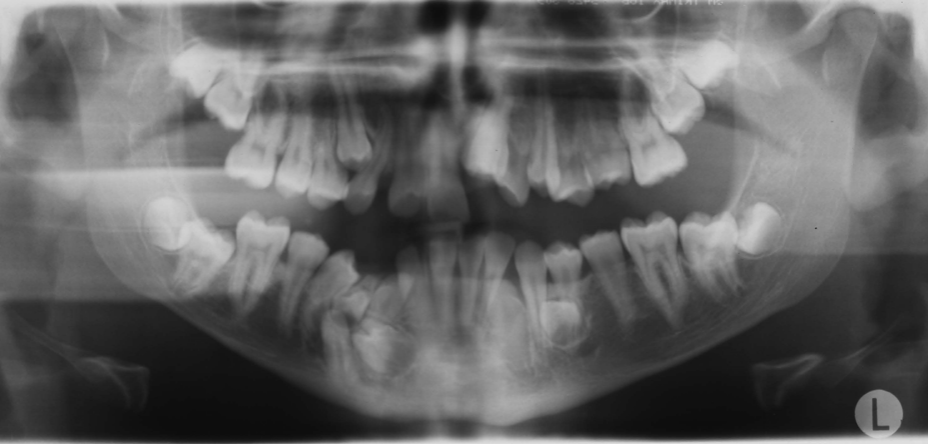
**Orthopantomography at 14 years 1 months of age**.

**Figure 4:**
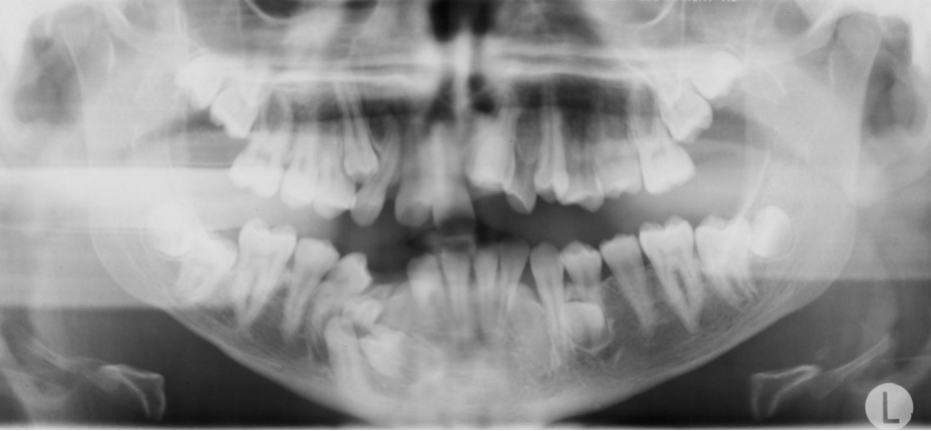
**Orthopantomography at 14 years 6 months of age**.

A 15-year-old man, with a diagnosis of Cleidocranial dysplasia, was referred to our department for orthodontic treatment. The patient was previously treated only with deciduous and supernumerary teeth extraction and four panoramic x-ray were taken in the last two years in order to check teeth development and eruptive direction (Figure 1, 2, 3, 4).

### Oral examination revealed a bilateral cross bite with marked teeth misalignment

Old radiograms examination shows the presence of several supernumeraries and impacted teeth: we were able to estimate the number of impacted teeth but it was not possible to exactly evaluate their morphology, in order to detect possible root reabsorption areas and to establish which were supernumeraries teeth and which not, and their anatomical relationship with other teeth and with some critical structures like mandibular nerve and foramen.

On the basis of these findings a three-dimensional CBCT scan was obtained, in order to exactly recognize teeth anatomical anomalies, to decide which of them are to be extracted and to plan the surgical access.

We decided to use the NEWTOM 3G (QR, Verona - Italy) scanner and we choose the spherical field of view (FOV) of 9" (medium FOV) in order to obtain an image of only maxillary, mandibular and TMJ region with a unique acquisition. The scan was completed within 36 seconds and the time of exposure was of 3,6 seconds. The tube voltage was of 110 kV and the tube current was of 4,70 mA.

**Figure 5:**
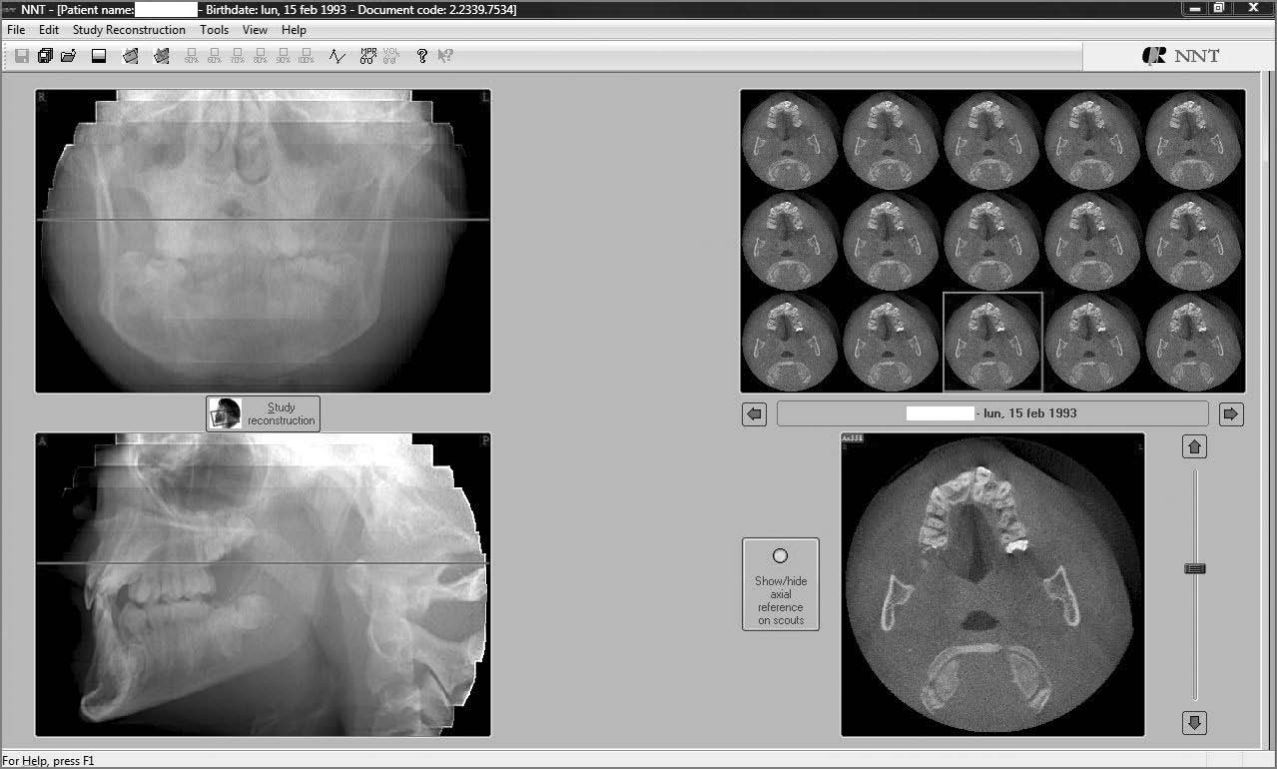
**An axial section of the maxilla showing the relationship between the upper central left incisor and a supernumerary tooth**.

We used the 2.11 version of the QR NNT program to visualize the most interesting axial sections showing the impacted and the supernumerary teeth (Figure 5).

**Figure 6:**
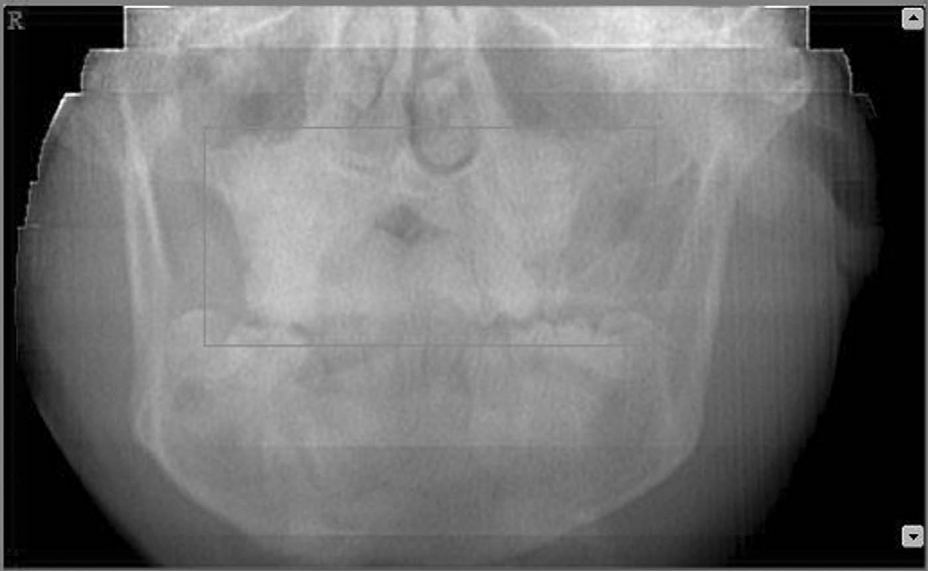
**Selection of the area to be studied - Frontal view**.

**Figure 7:**
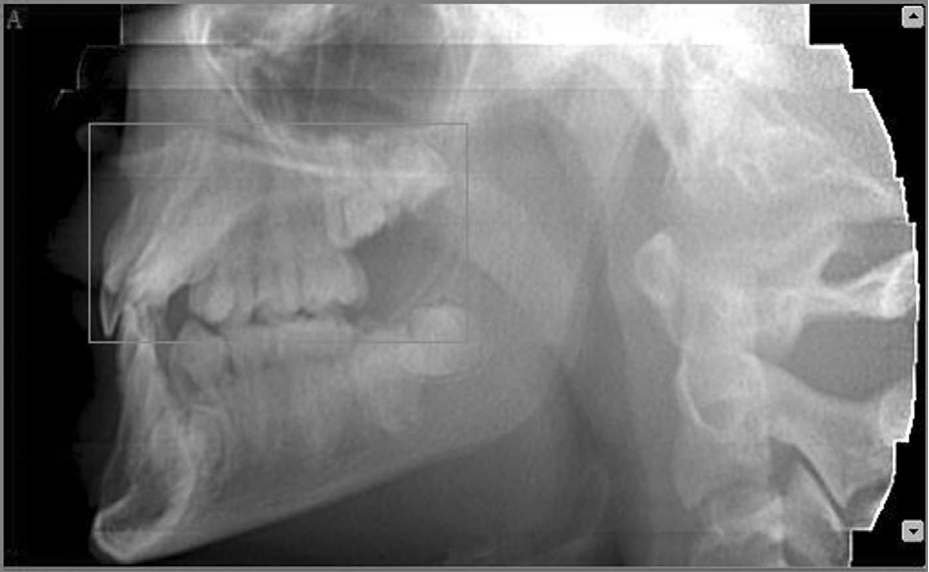
**Selection of the area to be studied - Sagittal view**.

**Figure 8:**
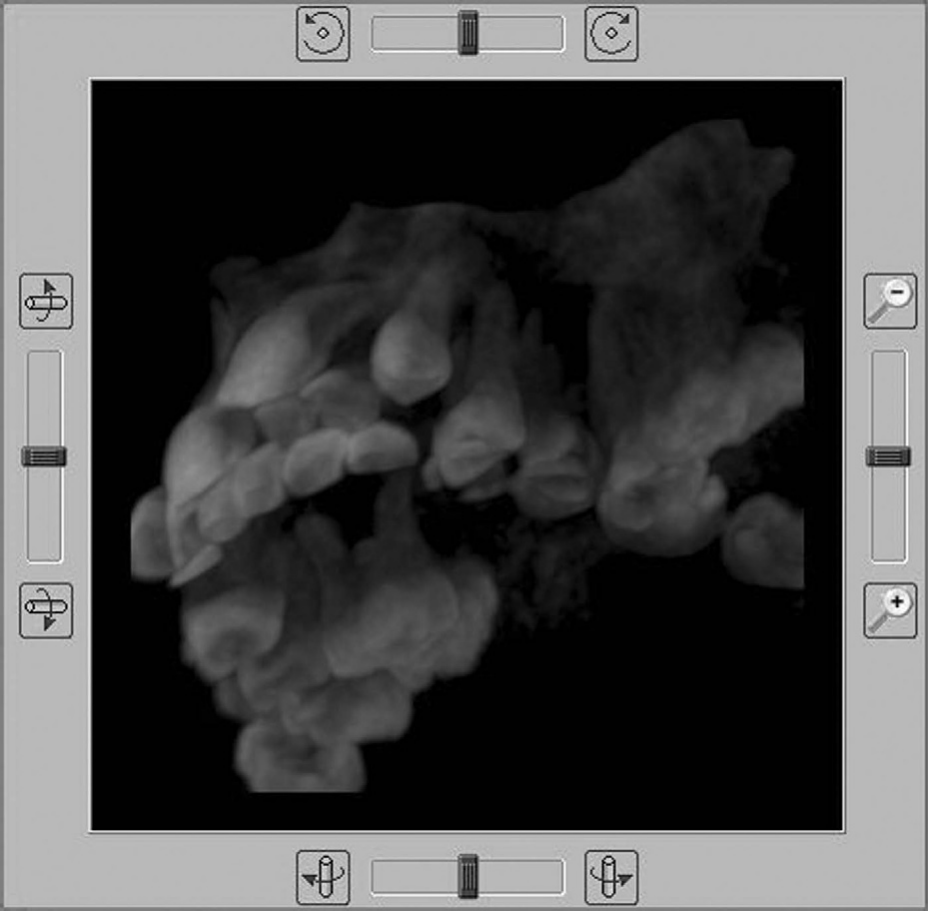
**Sagittal section of the maxillary arch displayed in MIP mode: bottom left side view**.

Afterward we selected a limited area of the volume acquired (Figure 6, 7) and we utilized the study reconstruction function to prepare a 3D image of this volume, that can be rotated in all the directions to better visualize every possible perspective (Figure 8).

**Figure 9:**
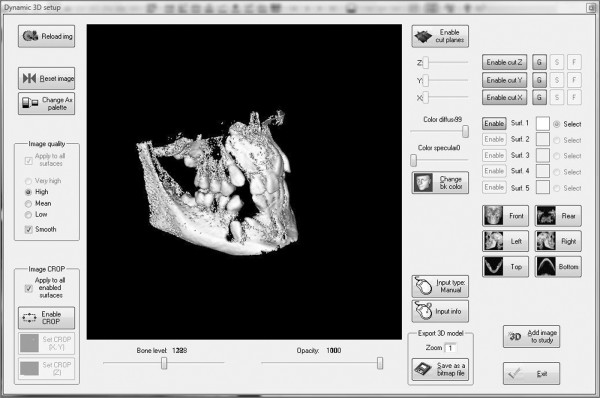
**3D dynamic setup of the maxilla-mandibular complex: front right side view**.

We also utilized the dynamic 3D function in order to obtain a panoramic overview of the intermaxillary dental relationship (Figure 9).

**Figure 10:**
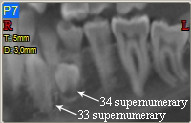
**Panoramic view of left mandible**.

**Figure 11:**
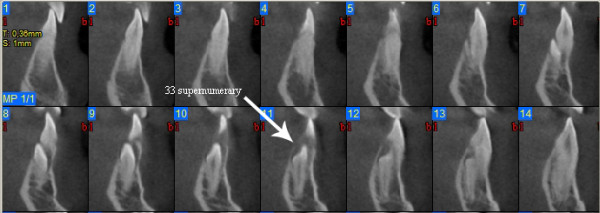
**Cross sections at 3.3 supernumerary level**.

**Figure 12:**
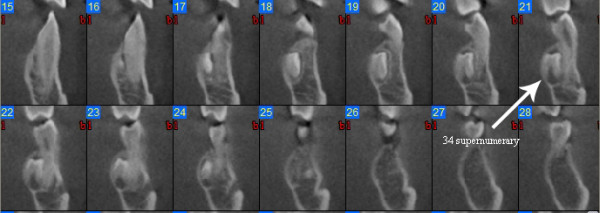
**Cross sections at 3.4 supernumerary level**.

**Figure 13:**
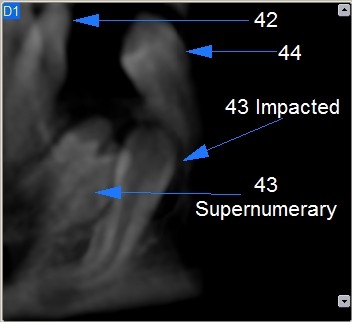
**4.3 impacted and supernumerary lingual view**.

**Figure 14:**
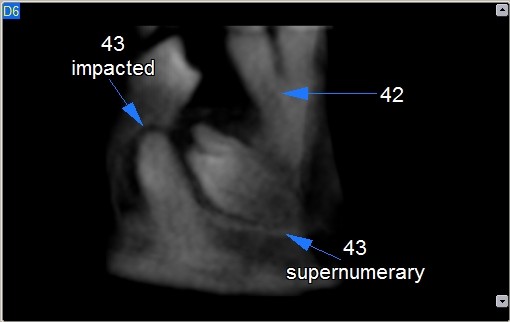
**4.3 impacted and supernumerary vestibular view**.

**Figure 15:**
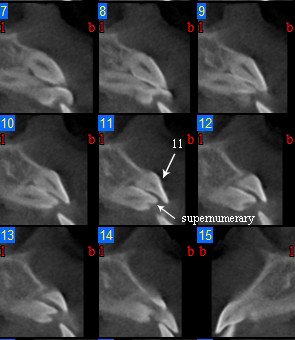
**Cross sections at 1.1 supernumerary level**.

Images analysis showed the presence of three supernumeraries - between 3.2 and 3.3 (Figure 10, 11), 3.3 and 3.4 (Figure 10, 12), 4.4 and 4.2 (Figure 13) - and one impacted tooth - 4.3 (Figure 14) - in the mandible, one supernumerary in the maxilla in 1.1 position (Figure 15).

Sections from 6 to 14 in figure nr. 7 show that 3.3 supernumerary is positioned lingually between tooth 3.3 and tooth 3.2, that are inclined due to the presence of this supernumerary but with no sign of root reabsorption: surgical approach will be from the lingual side, paying attention to respect roots integrity of teeth 3.3 and 3.2, that are very close to the supernumerary.

Sections from 21 to 24 in figure nr. 8 show the relationship between supernumerary 3.4, tooth 3.4 and mental foramen. The supernumerary is situated lingually to tooth 3.4 and there is no contiguity with mental foramen, so its extraction could be performed safely with a lingual surgical approach; tooth 3.4 is buccally displaced, resulting in a thin cortical buccal bone, and there is a root reabsorption at the limit between the medial and the coronal third of the root: considering these findings treatment decision will consist in supernumerary 3.4 extraction and lingual repositioning of tooth 3.4 root, keeping in mind that, depending on root reabsorption, long term prognosis of this tooth is uncertain.

Figures nr. 9 and 10 analysis shows that impacted 4.3 root is curved at the apical half: this could make more difficult or maybe even to block its orthodontic forced eruption.

Figure nr. 11 shows that supernumerary 1.1 extraction could be performed easily with no risk of damaging tooth 1.1.

## Discussion

The use of 3D computer assisted tomography is the best, and probably the only one, method permitting to elaborate a real individual orthodontic treatment plan for each CCD patient. It allows to precisely locate the impacted or ectopic teeth and therefore to perform a minimally invasive surgery and to plan the most effective orthodontic strategies [17,18].

Therefore CT images permit to safely place titanium screw, that have been suggested by Kuroda [19] to be very useful as an absolute anchorage during the forced orthodontic traction of impacted teeth in order to reduce the patient's treatment time and psychological stress, in both maxilla and mandible, avoiding the risk of damaging during the screw insertion some important surrounding anatomical structures like dental roots, nerves and blood vessels.

The shortcoming of the routinely use of this technique was related to the high radiation exposure of the patient, limiting the application only at complex cases. All other cases were studied with traditional dental radiology using the tube shift method (parallax technique), that taking two conventional radiographs permit to locate the impacted tooth location comparing the movement of this tooth respectively to the way in which the radiograph was taken.

Rosenstein [20], using a technique suggested by Dado [21] for the study of the bone support of a tooth consisting on the visual inspection of a series of CT slices perpendicular to the root axis and the method of Bland and Altman [22] for assessing agreement of two different measures, found that traditional radiology is reliable in the cases where bone support is good or poor, but it's more inaccurate than a CT scan in the intermediate cases.

Ericson and Kurol [23] documented that, even if traditional radiology permit in most cases to locate an impacted tooth, it frequently underestimate the presence and the extension of root resorption of the adjacent teeth.

This dichotomy between conventional radiology and computed tomography can be overcome utilizing a CBCT system, that permit to obtain an accurate 3D reconstruction and several sagittal, frontal and axial view of the impacted tooth, with a radiation exposure level that lay between multi slice CT (MSCT) and conventional radiography [24]. Of course the radiation exposure of the patient always depends on the setting (kV, mA and seconds of administration) used during the radiological exam: therefore every comparison between "general systems", like CBCT vs conventional radiology or CBCT vs MSCT, can produce only relative and approximate results. In order to conduct a precise comparison it's necessary to specify all the setting machine data. For example Ludlow [25] compared several CBCT scanners, with different FOV, with an average panoramic dose that they found using a Planmeca Promax digital panoramic device: using a different panoramic device, especially if using a non digital one, would have led to different results.

**Figure 16:**
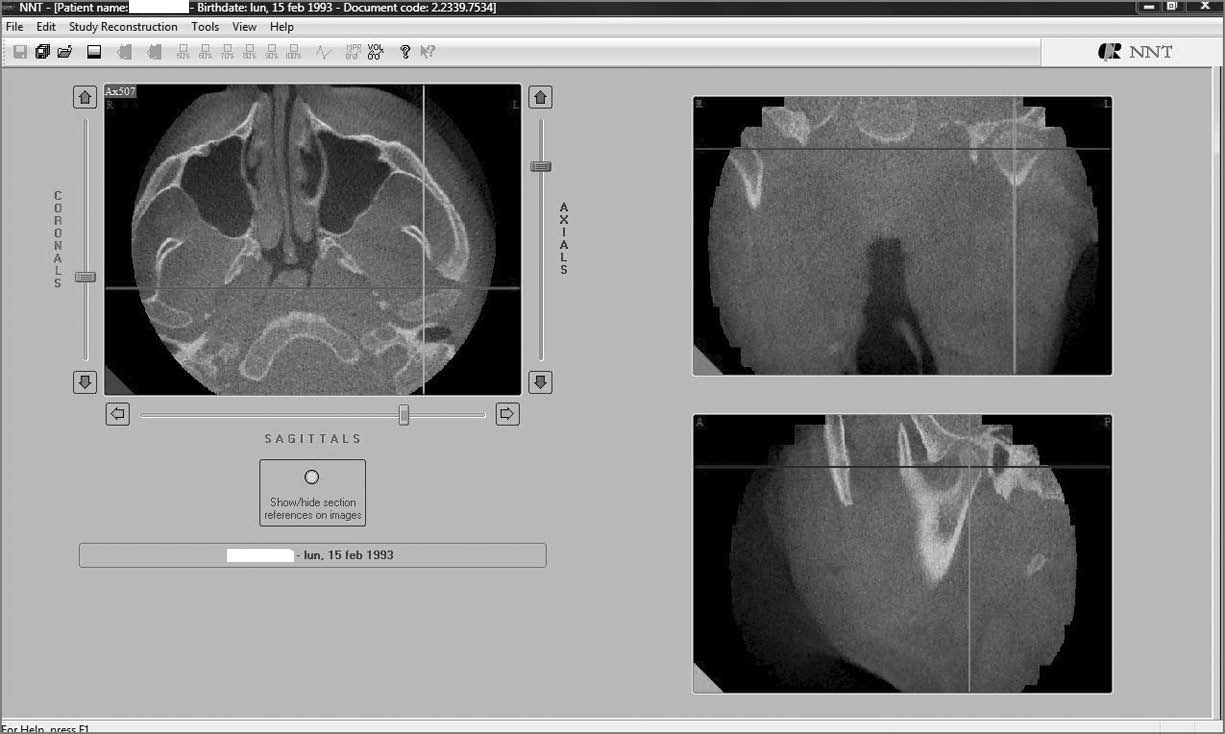
**Coronal, sagittal and axial view of the left condyle**.

**Figure 17:**
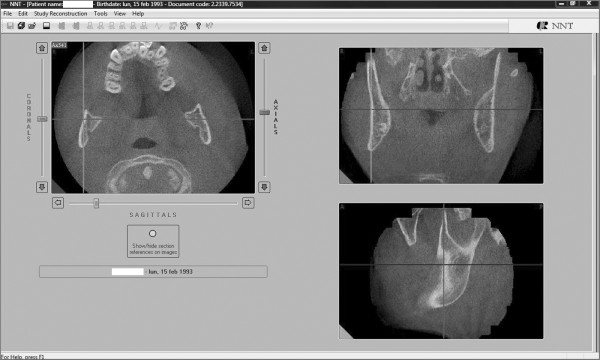
**Coronal, sagittal and axial view of the two mandibular ramus**.

Therefore we utilized in this case report the medium NewTom 3G FOV of 9" that, with an effective dose lower than the large FOV of 12" (indicated by Ludlow as administering a lower effective dose face to the most of the others CBCT scanners included in his study), permits to perform a three-dimensional study of: the upper airways [26]; the temporomandibular joint (TMJ) morphology (Figure 16), that is useful especially in these patients that are candidate to a an orthognatic surgery because of the risk of consequent condylar resorption [27]; the ramus (Figure 17), that has been described by McNamara [15] as typical with nearly parallel borders in CCD patients.

## Conclusion

In CCD patients the use of reconstructed 3D images obtained by a CBCT exam for diagnosis and treatment planning has only scarcely been documented until now, so no evidence-based conclusion can be made based on the current literature. CCD diagnosis is frequently made during the early childhood or even at birth, consequently it's incorrect to state that CBCT could be useful in identifying CCD. On the other hand, there is a general agreement that 3D images allows to obtain a more accurate reconstruction of the real anatomy than traditional 2D radiologic images, really useful especially in patients with impacted and supernumeraries teeth like CCD ones, even if there is no agreement about an extensive use of CT exams.

We present this case report to support the use of a less invasive CT exam, the low dose CBCT technology, in CCD patients in late mixed dentition undergoing orthodontic treatment and to promote the collection of sufficient data to come to a common agreement on the use of 3D radiological exam in these patients.

## Consent

Written informed consent was obtained from the patient for publication of this case report and accompanying images. A copy of the written consent is available for review by the Editor-in-Chief of this journal.

## Competing interests

The authors declare that they have no competing interests.

## Authors' contributions

All authors read and approved the final manuscript. DD conceived of the study, and participated in its design and coordination and helped to draft the manuscript. LL has been involved in drafting the manuscript and to collect the results from follow-up examinations. MP has been involved in revising the manuscript critically for important intellectual content. CP and PB have done substantial contributions to conception and design and interpretation of data.
